# Structural basis of DNA sequence recognition by the response regulator PhoP in *Mycobacterium tuberculosis*

**DOI:** 10.1038/srep24442

**Published:** 2016-04-15

**Authors:** Xiaoyuan He, Liqin Wang, Shuishu Wang

**Affiliations:** 1 Department of Biochemistry & Molecular Biology, Uniformed Services University of the Health Sciences, 4301 Jones Bridge Road, Bethesda, Maryland 20814, USA

## Abstract

The transcriptional regulator PhoP is an essential virulence factor in *Mycobacterium tuberculosis*, and it presents a target for the development of new anti-tuberculosis drugs and attenuated tuberculosis vaccine strains. PhoP binds to DNA as a highly cooperative dimer by recognizing direct repeats of 7-bp motifs with a 4-bp spacer. To elucidate the PhoP-DNA binding mechanism, we determined the crystal structure of the PhoP-DNA complex. The structure revealed a tandem PhoP dimer that bound to the direct repeat. The surprising tandem arrangement of the receiver domains allowed the four domains of the PhoP dimer to form a compact structure, accounting for the strict requirement of a 4-bp spacer and the highly cooperative binding of the dimer. The PhoP-DNA interactions exclusively involved the effector domain. The sequence-recognition helix made contact with the bases of the 7-bp motif in the major groove, and the wing interacted with the adjacent minor groove. The structure provides a starting point for the elucidation of the mechanism by which PhoP regulates the virulence of *M. tuberculosis* and guides the design of screening platforms for PhoP inhibitors.

Gene transcription regulation is of central importance in cell differentiation, cell-cell communication, and cellular responses to environmental cues. To perform this important function, transcriptional regulators must be able to recognize specific gene promoters by binding to specific DNA motifs. In bacteria, the adaptation to the environment at the transcriptional level is often mediated by a group of signalling proteins called two-component systems^1^. Each system typically consists of a histidine kinase and a response regulator (RR). The histidine kinase senses environmental signals that activate its kinase activity to phosphorylate the RR, which in turn regulates gene transcription to generate cellular responses. In *M. tuberculosis*, the response regulator PhoP of the two-component system PhoPR regulates the expression of more than 100 genes, including major secreted antigens such as Esat6^2^,^3^. The deletion of either the *phoP* or *phoR* gene in *M. tuberculosis* severely attenuates its virulence^4^,^5^,^6^,^7^, and attenuated strains are being developed as live vaccines^8^,^9^. Therefore, inhibitors that disrupt the PhoPR functions can be developed as drugs against tuberculosis (TB).

PhoP belongs to the OmpR/PhoB family of RRs, which is the largest family and contains thousands of proteins^10^,^11^. These RRs have two distinct domains: an N-terminal receiver domain (RD) that contains the conserved aspartate residue as the phosphorylation site and a C-terminal effector domain, also known as DNA-binding domain (DBD), that has a winged helix-turn-helix fold. Despite extensive research in recent years, the molecular mechanism of DNA sequence recognition by this large family of RRs is not fully understood.

All known DNA sequences that bind OmpR/PhoB family RRs are direct repeats, suggesting that these RRs bind DNA as tandem dimers. This tandem dimer association is observed in the crystal structure of the *E. coli* PhoB DBD-DNA complex^12^. However, the RDs are expected to form a symmetric dimer according to the structures of isolated RDs that are activated by binding to the phosphorylation mimic BeF_3_^−^. Most of these RDs dimerize through an interface involving α4-β5-α5 elements^13^,^14^. Additionally, some non-activated RDs form similar dimers^15^.

The crystal structure of full-length PhoP reveals that it can form a symmetric RD dimer involving the α4-β5-α5, with the DBDs of the dimer dangling by a disordered linker^16^. However, PhoP exists predominantly as a monomer in solution. These results led to the hypothesis that the phosphorylation of the RD promoted its dimerization and thus brought the two DBDs into close proximity to bind the DNA direct repeat^17^. This hypothesis appears to be consistent with the structure of the *Escherichia coli* KdpE-DNA complex^18^ and the *Klebsiella pneumoniae* PmrA-DNA complex^19^, which are currently the only available structures of full-length RR-DNA complexes in the OmpR/PhoB family. Both structures reveal a symmetric RD dimer that connects to a tandem DBD dimer. Because of differences in the structural organization between the RD and DBD, the structure is relatively open. This open structure cannot explain the highly cooperative binding of the PhoP dimer to the DNA and the tendency of the phosphorylated PhoP to form dimer, trimer, and higher-order oligomers^20^. Moreover, the strict requirement for a 4-bp spacer between the direct-repeat motifs of the PhoP recognition sequences suggests that the PhoP dimer is likely to have a strong interface that involves both the receiver and effector domains.

To uncover the mechanism by which PhoP binds DNA, we determined a crystal structure of PhoP in complex with its consensus-binding sequence. The structure reveals that a compact tandem dimer of PhoP binds to the DNA direct-repeat sequence. The compact dimer interface explains the highly cooperative binding of the PhoP dimer to the DNA direct repeat and the strict requirement for a 4-bp spacer. PhoP binds DNA through a positively charged surface matching the DNA phosphate backbone, specific interactions of the sequence recognition helix with bases of the DNA major groove, and the interaction of the wing with the minor groove. This structure can guide the design of inhibitor screening platforms. Furthermore, the mechanism underlying DNA sequence recognition likely applies to related transcriptional regulators.

## Results

### Overall structure of the PhoP-DNA complex

The PhoP-DNA complex was crystallized as a 2:1 complex consisting of two molecules of PhoP bound to one DNA duplex containing a direct repeat^20^. The crystal structure was determined to a resolution of 2.4 Å (Table 1). The smallest repeating volume of the crystal (i.e., the asymmetric unit) contains two PhoP-DNA complexes. These two complexes are not related by any rotational symmetry. Their two DNA duplexes are antiparallel to one another, and each forms a pseudo-continuous DNA double helix throughout the crystal by pairing the G/C overhangs with neighbouring molecules, thereby weaving the protein-DNA complexes into the crystal. The structures of two independent PhoP-DNA complexes are essentially identical (Supplementary Fig. S1).

In each PhoP-DNA complex, the two protein subunits assemble as a tandem dimer on the DNA direct repeat. Each subsite of the direct repeat binds one PhoP subunit and interacts with only the DBD. The PhoP-DNA interactions are identical at both subsites. The two DBDs of the PhoP dimer form a tandem head-to-tail arrangement similar to that observed in the PhoB-DNA^12^ and the KdpE-DNA^18^ structures. Surprisingly, the two RDs also associate in a tandem manner, in contrast to an earlier prediction of a symmetric RD dimer^16^,^17^.

**Figure 1:**
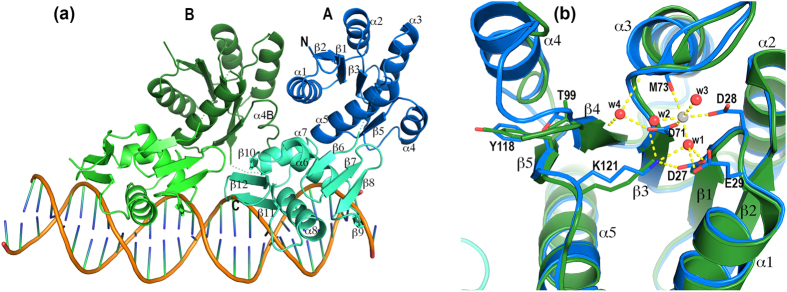
Structure of the PhoP-DNA complex. (**a**) Ribbon diagram of the PhoP-DNA complex showing a tandem PhoP dimer binding to a direct-repeat DNA sequence. The two PhoP subunits are designated A and B, with subunit A binding to the first TCACAGC motif of the direct repeat and B to the second motif. Each domain of PhoP is coloured individually as follows: blue and cyan for the subunit A RD and DBD, respectively, and dark green and light green for subunit B. Secondary structural elements are labelled on subunit A. Helix α4 is unwound in subunit B and is labelled α4B. The sequence-recognition helix α8 is inserted into a major groove to contact the bases in the TCACAGC motif, whereas the wing interacts with the downstream minor groove. Dotted lines indicate residues that are disordered in the crystal structure. (**b**) Structural alignment of the two subunits around the phosphorylation site. Subunit A is coloured in blue and B is in green. A Ca^2+^ ion is found near the phosphorylation acceptor Asp71 with hexa-coordination as follows: two ligands from the Asp71 and Asp28 side chains, one from the carbonyl of Met73, and three from ordered water molecules. Side chains involved in coordinating the Ca2^+^ ion are shown only for the subunit A. The side chains of Thr99, Tyr118, and Lys121 are shown for both subunits.

The tandem association of the PhoP receiver domains allows for compact dimer formation upon binding to the direct-repeat DNA (Fig. 1). The PhoP-dimer assembly involves both intra- and intersubunit domain interfaces. The intrasubunit RD-DBD interactions are identical in both PhoP subunits. The intersubunit interactions include not only the RD-RD and DBD-DBD interactions but also the RD-DBD interactions. The PhoP dimer buries 3776 Å^2^ of surface area (a total area of all domains calculated with AREAIMOL^21^ of the CCP4 Suite^22^) in the domain interfaces and has a total solvent-exposed surface area of 19971 Å^2^. The ratio of the buried surface area to the solvent-exposed surface area is 0.189. In comparison, the KdpE dimer (PDB ID 4KFC^18^) buries 3043 Å^2^ of surface area and has a total solvent-exposed surface area of 20960 Å^2^, a ratio of 0.145.

### Intrasubunit domain interface

The intrasubunit domain interface covers ~600 Å^2^ and involves helix α5 and its preceding loop in the RD and helices α6 and α7, the loop following α7, and the loop between strands β7 and β8 in the DBD (Fig. 1a). The RD and DBD interact through helix dipole interactions, hydrogen bonds, and hydrophobic, aromatic, and charge interactions. The N-terminus of α5 is adjacent to the C-terminus of α7 and contributes to helix-dipole interactions, a hydrogen bond between a backbone amide and a carbonyl, and a water-mediated main-chain hydrogen bond (Supplementary Fig. S2). The first turn of α5 interacts with α6 through charge-charge interactions, one hydrogen bond, and water-mediated hydrogen bonds. The C-terminal half of α5 interacts with the loop between β7 and β8 via water-mediated hydrogen bonds, charge and aromatic interactions, and hydrophobic interactions. The loop preceding α5 interacts with the loop following α7 through one main-chain hydrogen bond and side-chain aromatic interactions between residues Phe123 and Tyr205. These two residues are also involved in the intersubunit dimer interface, thereby linking the two types of interfaces.

### Intersubunit dimer interface

Interactions between the dimer subunits involve all domains and cover ~790 Å^2^ per monomer. Because the subunits are arranged in tandem, the two PhoP subunits have different environments. To differentiate between the two subunits, we will refer to the upstream subunit that binds to the first TCACAGC motif as molecule A and the downstream subunit as molecule B (Fig. 1). The dimer interface can be described as two separate patches as follows: a major patch involving both domains of molecule A, which cradle receiver domain B, and a minor patch involving the two DBDs only.

**Figure 2:**
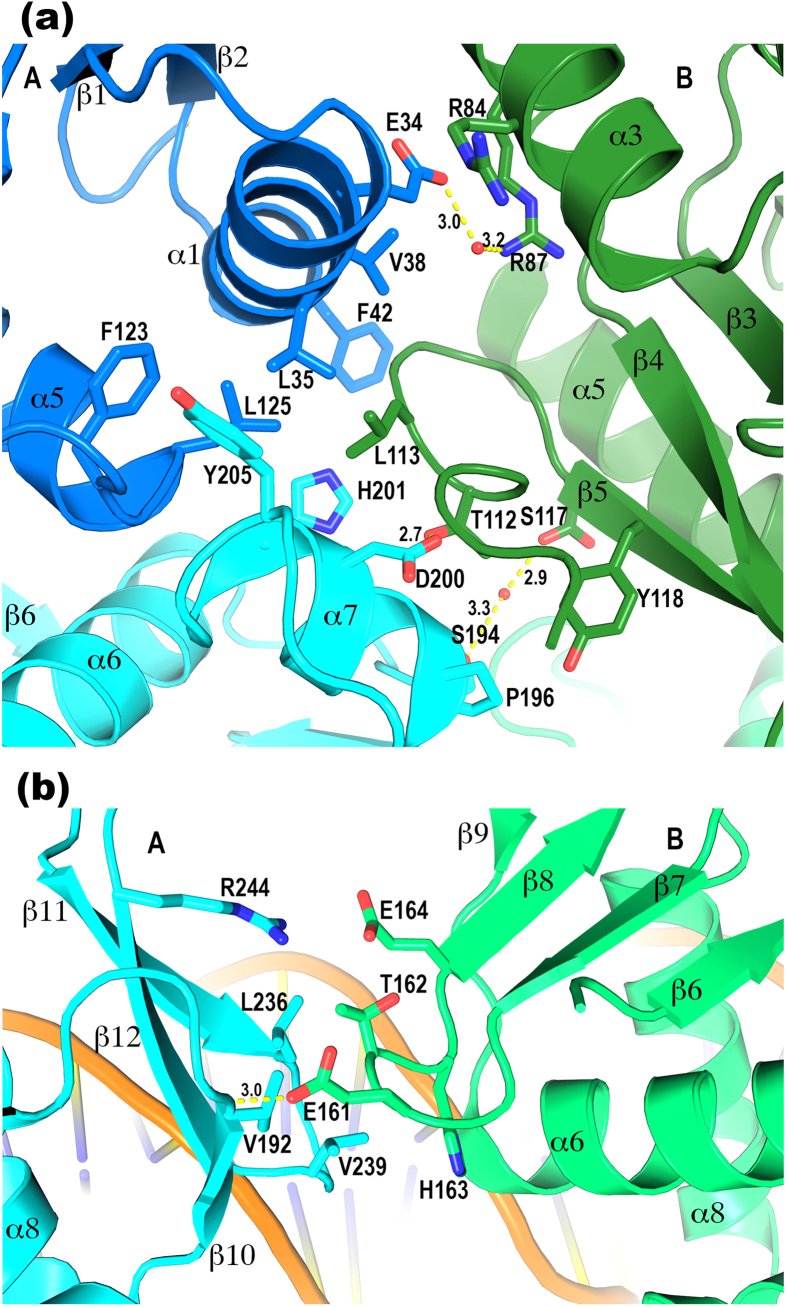
PhoP tandem dimer interface. (**a**) The major patch of the dimer interface where both domains of subunit A interact with the RD of subunit B. The colour scheme is identical to that in Fig. 1. The side chains involved in the dimer interface are shown as sticks. Hydrogen bonds are shown as yellow dashed lines with the distances labelled in Å units. The Leu113 in B has hydrophobic interactions with the Leu35, Leu125, and His201 in A. The Tyr118 in B has hydrophobic interactions with the Pro196 in A. The Phe42 side chain stacks with the peptide plane of Gly114, and the Arg84 guanidinium group stacks with the peptide plane of Asn31 at the N-terminus of α1, both at a distance of ~4 Å. (**b**) The minor patch of the dimer interface where the DBDs of the two subunits meet. The RD of A is coloured in cyan and B is in light green. A hydrophobic patch in A consisting of the side chains Val192, Leu236, and Val239 interacts with the hydrophobic parts of the Glu161, Thr162, and His163 in B.

The major patch primarily involves residues from helices α1 and α7 of A and α3 and α4 of B. Helices α3 and α4 exhibit large shifts between the two subunits of the PhoP dimer (Fig. 1b), indicating their high mobility and flexibility. Helix α4 of molecule B is located at the centre of the major patch of the dimer interface. This sequence segment is highly flexible and forms a one-turn helix in the DNA-free structure^16^ and a 1.5-turn helix in molecule A of the PhoP-DNA complex. However, the helix is unwound in molecule B and the loop preceding the segment is disordered. The side chain of Leu113 at α4 (we will refer to this structural segment as α4 even though the helix is unwound) sticks into a shallow hydrophobic pocket of molecule A (Fig. 2a). This hydrophobic pocket is primarily composed of side chains of the RD and is connected to the hydrophobic core of the domain. The pocket extends to the DBD to include Tyr205.

Additional interactions at the major patch of the interface include a hydrogen bond from Asp200 of α7 in molecule A to Thr112 of molecule B, hydrophobic interactions between the side chains of Pro196 (A) and Tyr118 (B), and π-π stacking of Phe42 (A) with the Gly114-Gly115 peptide bond (B). Arg84 and Arg87 of α3 (A) interact with α1 (B) through charge interactions, water-mediated hydrogen bonds, and π-π stacking.

The minor patch of the intersubunit interface occurs between the two DBDs (Fig. 2b). An exposed hydrophobic patch of the C-terminal β-sheet of molecule A is packed against loop β7β8 of molecule B through the hydrophobic interactions. There is also a hydrogen bond between the Glu161 side chain in A and the amide group of Val192 in B and a charge attraction between Arg244 (B) and Glu164 (A).

### Phosphorylation site and switch residues

The two RDs superpose very well with the exceptions of helices α3 and α4, which have different contacts in the two PhoP subunits (Fig. 1b). A metal ion is bound near phosphorylation acceptor Asp71. This cation is modelled as Ca^2+^ because Ca^2+^ is present in the crystallization buffer. The switch residues Thr99 and Tyr118 have inactive conformations, their side chains facing away from the phosphorylation site. As mentioned above, Tyr118 is involved in the dimer interface. Both Tyr118 and Thr99 interact with helix α4, and changes in their conformations can impact the position and conformation of this flexible helix, thereby modulating the dimer interface.

### PhoP-DNA binding interactions

**Figure 3:**
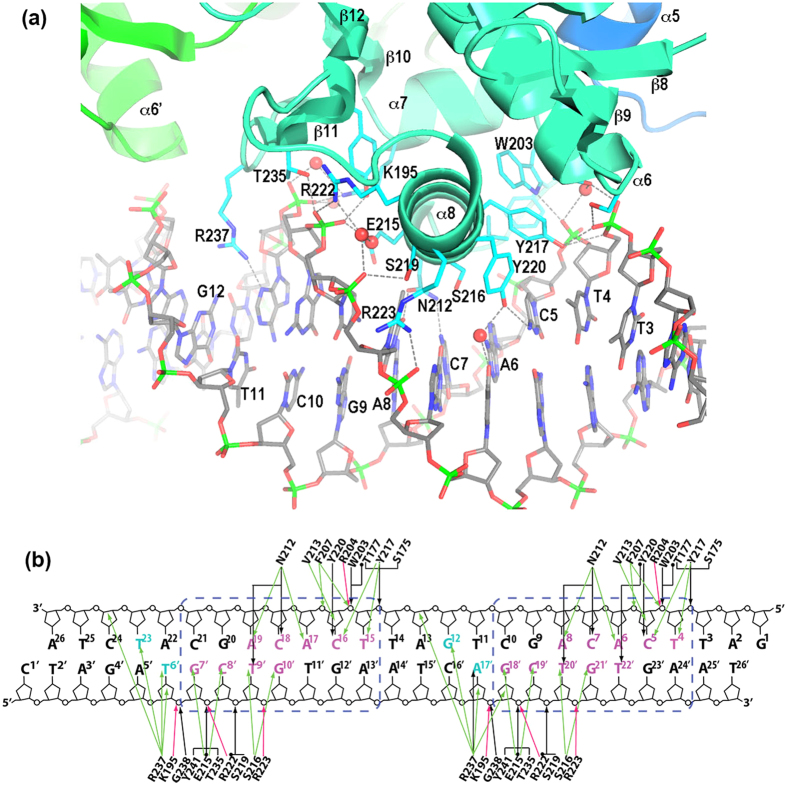
Details of PhoP-DNA interactions. (**a**) A ribbon diagram showing the DNA-binding elements of PhoP interacting with DNA. DNA is shown in sticks. The bases in the strand containing the TCACAGC motif are labelled. The side chains of PhoP that interact with DNA are shown as sticks. The hydrogen bonds between the protein and DNA are shown as dashed lines. (**b**) Schematic diagram showing detailed interactions between PhoP and DNA in both subsites of the direct repeat. DNA sequences of both strands in the crystal structure are shown. The base pairs of the TCACAGC motifs are boxed in blue dashed lines. Bases in the major groove that are in contact with protein atoms are coloured magenta; those in the minor groove in contact with the Arg237 side chain are coloured cyan. The hydrogen bonds are shown as black arrows, and water-mediated hydrogen bonds are marked with a dot at the beginning of the arrows. Van der Waals contacts within 4 Å are shown as green arrows, and salt bridges are shown as red arrows.

The PhoP-DNA interface exclusively involves the DBD and covers 1738 Å^2^ of the surface area of the PhoP dimer. The PhoP and DNA surfaces are complementary to each other in both contour and electrostatic potential (Supplementary Fig. S3). The positive charges on the PhoP surface match the DNA phosphate backbone, thereby contributing charge attraction to the binding affinity. Also contributing to the binding are aromatic and hydrophobic side chains on or near sequence recognition helix α8 that interact with the DNA phosphate and ribose groups, and the wing that interacts with the minor groove. Sequence-specific interactions are exclusively from sequence-recognition helix α8 to the DNA bases in the major groove. The PhoP-DNA interactions are essentially identical at both subsites of the direct repeat (Figs 1 and  3). Minor differences between the two subsites are due to the limited resolution of the crystal structure or variations in the DNA sequence outside of the TCACAGC motifs.

Next, we will describe the sequence-specific interactions at the upstream subsite with PhoP molecule A. Helix α8 packs tightly into the major groove and directly contacts the base pairs of the TCACAGC motif (Fig. 3a). Outward-facing side chains of α8 interact with the major groove by forming hydrogen bonds and aromatic, hydrophobic, and van der Waals interactions to recognize the DNA sequence. These side chains include Asn212, Val213, Glu215, Ser216, Tyr217, and Tyr220^23^.

Asn212 is located at the N-terminus of α8; its side chain is positioned in the middle of the major groove and contacts four bases (Fig. 3). Asn212 forms hydrogen bonds with C^7^ and T^20′^, van der Waals interactions with A^8^ with a distance of ~3.7 Å, and a water-mediated hydrogen bond with A^6^ (the bases are numbered from the 5′ end with superscripts 1 to 26 for the strand containing the TCACAGC motifs and 1′ to 26′ for the complementary strand, see Fig. 3b). The same water molecule that mediates the hydrogen bond to A^6^ also forms a hydrogen bond with the Ser216 side chain, which also resides in the middle of the major groove and is ~3.9 Å from T^20′^, thereby allowing favourable van der Waals interactions. Additionally, the Asn212 side chain has a hydrogen bond with the Glu215 side chain, which has aromatic interactions with C^19′^ (~3.4 Å) and G^18′^ (~3.8 Å).

Towards the 5′-end of the T^4^CACAGC motif, the Val213 side chain has van der Waals interactions with C^5^ at a distance of ~4.4 Å. The Tyr217 side chain has aromatic interactions with both C^5^ and T^4^. The Tyr220 side chain sits at the centre of the major groove and has a hydrogen bond with C^5^ and a water-mediated hydrogen bond with T^22′^. The same water molecule has a hydrogen bond with another water molecule that has a hydrogen bond with G^21′^ (pairing with C^7^). Overall, both bases of the middle 3 bp are in contact with protein side chains, whereas the 2 bp on each side have only one base that contacts the protein.

Nonspecific interactions in the major groove occur from residues of α8 and its vicinity to the backbone phosphates and ribose groups. Some of these residues are also involved in sequence-specific interactions, such as Glu215, Val213, and Tyr217. Interactions with phosphates primarily involve charged, polar, and aromatic side chains. On one side of the major groove, Arg222, Arg223, and Lys195 form salt bridges to the phosphate groups. The Ser219 side chain, which is mutated to Leu in the avirulent H37Ra strain^3^,^6^,^24^, has a hydrogen bond with the T^20′^ phosphate, and the Tyr241 side chain has a hydrogen bond and aromatic interactions with the C^19′^ phosphate. On the other side of the major groove, the aromatic side chains of Tyr217, Trp203, and Phe207 interact with the charges and π electrons of the phosphates. The Ser175, Thr177, Tyr217, and Trp203 side chains form hydrogen bonds, and the Arg204 forms a salt bridge with the phosphates. In addition to these interactions with phosphates, the Tyr217 and Val213 side chains have van der Waals interactions with the ribose groups. These nonspecific interactions contribute to the binding affinity and influence sequence-specific interactions by changing the conformation of the protein, DNA, or both.

### Interactions of the minor groove with the wing residues

Structurally, the wing (C-terminal β hairpin) is located adjacent to sequence-recognition helix α8 and interacts with the downstream minor groove (Figs 1 and  3a). The wing of molecule A interacts with the spacer sequence between the two 7-bp motifs, whereas the wing of molecule B interacts with the 3′ extension of the second motif. The Arg237 side chain of the wing inserts into the minor groove. Because the two Arg237 side chains contact different sequences, their interactions with the bases are slightly different (Fig. 3b). In the spacer minor groove, the Arg237 side chain has a hydrogen bond with A^17′^ and aromatic interactions with the bases of A^17′^ and G^12^. In the 3′-end minor groove, the Arg237 side chain has a slightly different orientation and no hydrogen bond with the bases. The interactions with the DNA backbone are similar for both subsites: van der Waals interactions with ribose groups and electrostatic attractions to the phosphates (Fig. 3a). In addition to Arg237, the main-chain amide of Gly238 and the side chain of Thr235 have hydrogen bonds with the phosphates. The wing plays an important role in the binding affinity and the limited sequence preference at the spacer and 3′ extension of the direct repeat^20^.

### Role of the domain interface in the cooperative binding of the dimer

To verify that the dimer interface observed in the crystal structure is relevant to PhoP functions, we mutated two interface residues as follows: Leu113 to Asp (L113D) and Tyr205 to Ala (Y205A). The effects of both mutations on binding affinity are similar and correlate with the binding affinities (Table 2). For the perfect direct repeat, which has a high affinity to PhoP, the mutations reduce the affinity slightly (statistically insignificant, p values > 0.14). The reduction in affinity is greater for weaker binding sequences. The sequence derived from the promoter of *hisG* has a K_d_ of 35.6 nM for the wild-type PhoP, and the K_d_ increases ~3-fold for the mutants. The sequence from *ahpC* binds PhoP more weakly with a K_d_ of 103.8 nM, and the mutations increase the K_d_ ~6-fold. The even weaker sequence from *lpqA* has a K_d_ of 347 nM for the wild-type PhoP and ~9-fold increase of K_d_ for the PhoP mutants.

**Figure 4:**
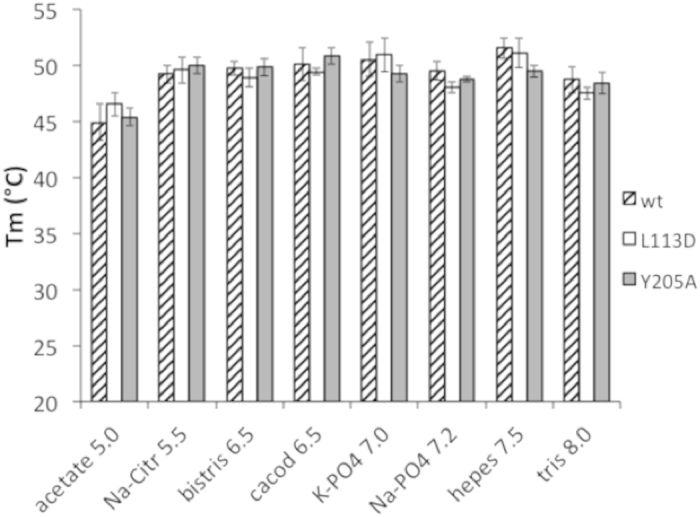
Melting temperature profiles of PhoP and its mutants. The buffers have a 100 mM concentration with the given pH, and they were adjusted with NaOH or HCl. Each buffer also contains 100 mM NaCl and 5 mM MgCl_2_. Error bars represent the standard deviations of 3–5 measurements. Melting temperatures of the PhoP wild type (wt) and the L113D and Y205A mutants were measured by Thermofluor as described in the text.

To assess whether the above reduction in affinity is caused by the lower stability of the mutants, we measured the melting temperatures of the proteins under various buffer conditions using Thermofluor^25^. The melting temperature profiles of the mutants are similar to those of the wild type (Fig. 4), suggesting that the mutations do not change the intrinsic stability of the protein.

As described above, the Leu113 of molecule B is at the centre of the major patch in the dimer interface (Fig. 2). Structural alignment indicates that, if PhoP forms a symmetric RD dimer through the α4-β5-α5, the L113 side chain is away from the dimer interface (Supplementary Fig. S5). Therefore, mutation of the residue is not expected to affect the symmetric RD dimer. The Tyr205 side chain is involved in both the dimer interface and RD-DBD interface (Fig. 2). Because the RD-DBD interface is part of the interfaces that form the tandem dimer and it is absent in the structure of PhoP alone^16^, disruption of this interface will destabilize the dimer. Therefore, our data indicate that the tandem-dimer interface is relevant to PhoP functions and that the weaker binding DNA sequences rely more on strong PhoP dimerization for cooperative binding.

### Contribution of the receiver domain to DNA binding affinity

The RD plays an important role in the cooperative binding of the PhoP dimer. The isolated effector domain (PhoPC) has a K_d_ of 188 nM (Table 2) in comparison with the K_d_ of ~19 nM for binding the full-length PhoP to perfect direct-repeat sequences. Similar to PhoP, the binding of PhoPC to the direct-repeat sequence as a dimer is highly cooperative. The K_d_ for PhoPC binding to half of the direct repeat (RD6-half in Table 2) is ~12 μM, which is more than 60-fold higher than the K_d_ for dimer binding to the direct repeat.

### Effect of the spacer length on PhoP-binding affinity

For the two PhoP subunits to maintain the dimer interface observed in the crystal, the two motifs must be separated by 4 bp. A shorter or longer spacer would change the relative position of the two binding sites, and as a result, the PhoP dimer interface observed in the crystal structure would be broken. This disruption would abolish the cooperative binding of the PhoP dimer and reduce the binding affinity. We analysed the binding of sequences with 2, 5, and 6 bp between the motifs, and we found that the binding affinity was significantly reduced compared with sequences with a 4-bp spacer^20^. To ascertain the strict requirement for the spacing, we measured the binding affinity of a direct repeat with a 3-bp spacer (Table 2) and found that the binding affinity was reduced by ~20-fold. Interestingly, mutant L113D did not exhibit any further reduction in binding affinity, suggesting that the dimer structure observed in the crystal structure did not support cooperative binding to the direct repeat with a 3-bp spacer.

### Binding of a PhoP tandem trimer and tetramer to DNA sequences

The tandem dimer observed in the crystal structure suggests that PhoP can potentially form higher-order oligomers by stacking in series. To determine whether such oligomers can form, we measured the PhoP binding affinity with sequences containing three motifs (tri) and four motifs (tetra) in a row with a 4-bp spacer in between the motifs. Both the tri and tetra sequences bound PhoP with a higher affinity than the direct repeat (Table 2), suggesting that the cooperative binding resulting from the tandem association of PhoP can be extended beyond a PhoP dimer. This finding is consistent with the tandem dimer assembly of PhoP on binding to DNA. The sequences with tri- and tetra-repeats are present in some gene promoters. In addition, phosphorylation of PhoP promotes the formation of dimer, trimer and higher-order oligomers; and the phosphorylation increases the DNA-binding affinity but does not alter the specificity^20^. Together, these observations suggest that tandem dimer assembly on the DNA direct repeat is relevant to the physiological function of PhoP.

## Discussion

PhoP represents a novel drug target because of its role in *M. tuberculosis* virulence. We showed that it was bound to a DNA direct repeat as a tandem dimer. The compact dimer interface explains why the dimer binds DNA in a highly cooperative manner. Although PhoP is a monomer in solution, it binds DNA only as a dimer^16^,^20^. This highly cooperative binding requires the two PhoP subunits to interact strongly and favourably upon DNA binding. Compared with the structures of KdpE-DNA and PmrA-DNA complexes^18^,^19^, the PhoP-DNA complex is more compact with a larger ratio of buried versus solvent-exposed surface area. The buried interface area excludes ordered water molecules around the protein and thereby contributes the favorable entropic effect to DNA binding.

The tandem dimer structure also contributes the favorable enthalpic effect by allowing both subunits to have optimal interactions with DNA. In the KdpE-DNA structure (PDB ID 4KFC), the downstream protomer (KdpE_B_) has a DNA-contact surface of 749 Å^2^, which is slightly less than that of KdpE_A_ of 794 Å^2^, and the wing of KdpE_B_ barely touches the minor groove whereas that of KdpE_A_ inserts into the minor groove. The differences are likely due to that the symmetric RD dimer puts the RD of KdpE_B_ away from the DNA, thereby pulling its DBD away from its optimal interactions with DNA. In comparison, the two PhoP subunits have identical interactions with DNA, and their contact areas with DNA are virtually identical (870 Å^2^ for A, and 874 Å^2^ for B).

Many other members of the OmpR/PhoB family are likely to share the same tandem dimer binding to DNA. These RRs bind to DNA sequences with a remarkably similar pattern. For instance, the DNA sequence motif for *Streptomyces coelicolor* PhoP binding is GTTCACCN_4_GTTCACC^26^, the *pho* box DNA sequence for *E. coli* PhoB binding is CTGTCAT(A/T)_4_CTGTCAT^27^, and the consensus sequence for the PhoPs of *E. coli* and *S. enterica* is TGTTTAN_5_TGTTTA^28^,^29^. One noticeable feature of these sequences that is shared by the consensus motif for the *M. tuberculosis* PhoP is that the equivalent bases of the repeated motifs have 10 bp spacing, which is the distance of one turn of the standard B-form of the DNA double helix. Another shared feature is that these RRs are present in solution as monomers but bind DNA only as dimers (i.e., highly cooperative dimer binding). These data strongly suggest that these RRs bind DNA as a compact tandem dimer, as observed for the *M. tuberculosis* PhoP.

The 10-bp spacing of equivalent bases is important for PhoP functions *in vivo*, as confirmed by the sequence motif derived from ChIP-seq studies^30^,^31^. This spacing puts the two PhoP-binding sites on the same side of the DNA double helix and thus allows the four domains of the protein to interact as observed in the crystal. Shorter or longer spacing would change the relative positions of the two binding sites and, as a result, they would alter the PhoP dimer interface. This phenomenon has been shown to occur in solution. Even a 1-bp insertion or deletion in the spacer dramatically reduces the binding affinity, suggesting that the cooperative binding is compromised. With the loss of cooperativity, the binding of PhoP to a direct-repeat sequence with a 3-bp spacer is not affected by the mutation at the dimer interface (Table 2). Moreover, the cooperative binding can be extended to trimer and tetramer binding to DNA, with proper repeats. Taken together, these results suggest that PhoP tandem-dimer assembly on the DNA is most likely the functional form of the protein and its closely related homologues.

PhoP binding to the direct repeat with strict 4-bp spacing is a mechanism for increasing the binding specificity. As described above, the sequence recognition helix interacts more weakly with the peripheral base pairs than it does with the three centre base pairs of the 7-bp motif, thereby potentially allowing many variations in the motif. Although a motif with various mismatches can be nearly ubiquitous throughout the genome, a direct repeat with strict spacing should be relatively sparse and thus greatly improves the binding specificity of PhoP. Many DNA-binding proteins use this strategy of a direct or inverted repeat with strict spacing to select a limited number of binding sites, thus achieving the desired specificity.

In conclusion, the response regulator PhoP recognizes DNA direct repeats on gene promoters by binding to DNA as a tandem dimer. The compact structure of the tandem dimer begets highly cooperative dimer binding and the strict requirement for a 4-bp spacer between the direct repeat motifs. The ability of PhoP to form a compact tandem dimer is important for the binding affinity and specificity and thus for the function of PhoP as a transcriptional regulator. This structure can guide the design of a platform for the high-throughput screening of small molecule inhibitors of PhoP-DNA binding. For example, by labelling the protein and DNA with fluorophores on locations guided by the structure, the Forster resonance energy transfer can be measured in the presence of a small molecule library. The mechanism by which PhoP binds as a compact tandem dimer to DNA and recognizes direct repeats with strict spacing most likely applies to many RRs of the same family. Finally, the mechanism by which the sequence recognition helix reads the DNA sequence at the major groove likely applies to transcription factors that bind DNA by inserting a helix into the major groove in an optimal manner to achieve the highest DNA sequence selectivity and binding affinity. The relative mismatch tolerance and the size of the sequence motif should be moderately conserved because they are constrained by the geometry of the protein helix and the DNA major groove. Interactions between an α-helix and a DNA major groove cover ~7 bp with better conservation of the central positions than the peripheral positions. This information should guide the *in silico* search for potential transcription factor-binding sites throughout whole genomes.

## Methods

### Site-directed mutagenesis

The *phoP* gene was mutated with a QuikChange II XL site-directed mutagenesis kit (Agilent Technologies, Santa Clara, California, USA) according to the manufacturer’s instruction. The pET28-*phoP*^16^ plasmid was used as the template, and the mutation primers were as follows: L113Df, gatcgcgggtctgaccGATggtggtgacgactatg; L113Dr, catagtcgtcaccaccATCggtcagacccgcgatc; Y205Af, cgaccacgtttggcgcGCcgacttcggtggtg; and Y205Ar, caccaccgaagtcgGCgcgccaaacgtggtcg.

### Protein expression and purification

The pET28-*phoP* plasmids containing the wild-type and mutant *phoP* genes, which encode proteins with an N-terminal His-tag that can be cleaved by the tobacco etch virus (TEV) protease, were transformed into *E. coli* strain BL21 (DE3). Protein expression was induced by adding IPTG. The proteins were purified by Ni^2+^-affinity column chromatography using a differential purification procedure^16^,^23^. In brief, the protein that was purified from the first Ni column was cleaved by the TEV protease to remove the His-tag and then passed through a second Ni column to separate the tag-free protein from the His-tag, the un-cleaved protein, and the His-tagged TEV protease. The proteins were further purified and buffer-exchanged with a Superdex 200 column (GE Life Sciences) prior to downstream applications. The purification of the isolated DBD of PhoP (PhoPC) was performed by following a similar procedure^23^.

### Protein crystallization, data collection and structure determination

The protein-DNA complex was prepared by mixing the protein with DNA at a 2:1 molar ratio in the binding buffer (20 mM HEPES, pH 7.5, 100 mM NaCl, and 5 mM CaCl_2_ or MgCl_2_) and purifying from a Superdex 200 column eluted with the same binding buffer. Crystals were grown using the microbatch method. Drops were set up by mixing 1 μl of the protein-DNA sample with 1 μl of the crystallization solution and covering them with a layer of 50% paraffin oil and 50% silicon oil. The best crystals were obtained from crystallization solutions containing 50 mM sodium cacodylate (pH 6.5), 10 mM CaCl_2_, 12% PEG 4000, and 2 mM spermine. Similar results were obtained from conditions containing MgCl_2_, CaCl_2_, or both. The highest resolution data set were obtained from a crystal grown in the presence of CaCl_2_ only.

The crystals were transferred to a cryo-solution containing 30% ethylene glycol in addition to the crystallization solution. The crystals were frozen in liquid nitrogen and tested for diffraction using an in-house X-ray system with a 007HF generator and a Raxis4++ detector (Rigaku). Well-diffracting crystals were transported to beamline X25 at the National Synchrotron Light Source, Brookhaven National Laboratory, for data collection with a Pilatus 6M detector. The best dataset had a high resolution at 2.4 Å (Table 1). The data were indexed and scaled with the HKL2000^32^. The crystals were in space group P2_1_2_1_2_1_, and they had 2 PhoP-DNA complexes per asymmetric unit and a Matthews volume of 2.62 Å^3^/Da.

The structure was determined by molecular replacement with PHASER^33^ using the RD of the full-length PhoP structure (3R0J)^16^ and the DBD-DNA complex of the *E. coli* PhoB (1GXP) as models^12^. The PhoB-DNA complex was separated into two halves, each containing one DBD with its associated DNA bases, to accommodate the difference in the DNA bending angle. PHASER identified 4 RDs and 4 DBD-DNA complexes. The initial electron density map revealed a continuous electron density connecting two DNA fragments and additional electron density at both ends to cover all residues of the DNA sequence (Supplementary Fig. S4).

Structural refinement was performed using REFMAC^34^. After each refinement cycle, the model was manually adjusted using COOT^35^. The last few refinement cycles were performed using BUSTER with TLS refinement^36^. The refinement statistics are reported in Table 1. Over 96% of the protein residues are in the preferred region of the Ramachandran plot as reported by the structure validation software MolProbity^37^.

### ITC Measurements

ITC experiments were conducted at 25 °C with a MicroCal iTC200 system in a buffer containing 20 mM HEPES (pH 7.5), 100 mM NaCl, and 5 mM MgCl_2_ as described previously^20^. The DNA sample in the syringe was titrated into the protein sample in the cell. The sample cell was stirred at 800 or 1000 rpm. The data were fitted using Origin 7.0 with a one-set-of-sites binding model.

### Size-Exclusion Chromatography

PhoP was mixed with double-stranded DNA fragments in binding buffer (20 mM HEPES, pH 7.5, 100 mM NaCl, and 5 mM MgCl_2_) at room temperature for 20 min. The protein-DNA mixture was loaded onto a Superdex 200 HR 10/30 column (GE Life Sciences) equilibrated with the binding buffer and eluted at room temperature at a flow rate of 0.5 ml/min.

### Thermofluor

The protein thermal melting curves were measured using the Thermofluor method^25^ with an ABI Prism 7900HT system. Protein at a ~0.02 mg/ml concentration was mixed with 1000x diluted SYPRO Orange (Life Technologies) in various buffers in a 96-well PCR plate. The samples were heated from 25 °C to 95 °C at a ramp rate of 1%, and the fluorescence intensity was measured continuously. The data were fitted to a Boltzmann model^25^ using EXCEL Solver (Microsoft Office) to obtain the melting temperatures.

## Additional Information

**How to cite this article**: He, X. *et al*. Structural basis of DNA sequence recognition by the response regulator PhoP in *Mycobacterium tuberculosis. Sci. Rep.* **6**, 24442; doi: 10.1038/srep24442 (2016).

**Accession codes:** Coordinates and structure factors have been deposited in the Protein Data Bank under the accession code 5ED4.

## Supplementary Material

Supplementary Information

## Figures and Tables

**Table 1:** Data collection and structural refinement statistics.

Data collection^a^	
Space group	P2_1_2_1_2_1_
Cell dimensions	
a, b, c (Å)	92.24, 98.18, 167.89
Resolution (Å)	30.0–2.40 (2.44–2.40)^b^
R_merge_ (%)	11.2 (71.2)
R_pim_ (%)	3.3 (27.9)
I/σ(I)	29.4 (2.13)
Completeness (%)	90.0 (54.2)
Redundancy	11.6 (6.1)
**Refinement**	
Resolution (Å)	30.0–2.40 (2.44–2.40)
No. reflections	55,261 (2,164)
R_work_/R_free_^c^ (%)	18.3/22.7 (22.3/30.3)
No. atoms	
Protein	6,945
DNA	2,120
Water	233
Ca^2+^ ions	6
Ethylene glycol	4
B factors (Å^2^)	
Protein and DNA	87.3
Water	74.9
Ca^2+^ ions	84.4
Ethylene glycol	87.4
Rmsd bond lengths (Å)	0.011
Rmsd bond angles (°)	1.11

^a^Data were collected at 100 K.

^b^Values in parentheses are for the highest-resolution shell.

^c^R_free_ was calculated with a subset of data (5%) that was never used in refinement.

**Table 2:** Effects of mutations at domain interface of PhoP and of the DNA sequences on PhoP-DNA binding measured by ITC.

Sequence	PhoP	Kd, nM	ΔH, kcal/mol	N^b^
DR^a^cgat**TCACAGC**tgat**TCACAGC**atctacg	wtY205AL113D	19.2 ± 2.821.8 ± 6.226.2 ± 6.5	−14.2 ± 0.1−18.2 ± 0.2−19.4 ± 0.1	0.460.510.50
*hisG*cgcgac**cCgCAGC**atgc**TCACAGC**tttcga	wtY205AL113D	35.6 ± 4.3114.5 ± 11.2116.6 ± 11.7	−14.5 ± 0.1−16.2 ± 0.1−15.8 ± 0.1	0.480.480.44
*ahpC*tttgcc**TgACAGC**gact**TCACgGC**acgatg	wtY205AL113D	103.8 ± 7.2653.6 ± 57.2595.2 ± 51.4	−13.0 ± 0.1−16.4 ± 0.3−15.1 ± 0.2	0.500.450.43
*lpqA*gcacct**TCACcGC**ccgc**cCACAGC**cacgca	wtY205AL113D	347.2 ± 35.13076.9 ± 371.12873.6 ± 540.9	−8.31 ± 0.08−13.8 ± 0.7−10.3 ± 0.9	0.510.430.39
DRs3^a^agtacc**TCACAGC**act**TCACAGC**tttcat	wtL113D	432.9 ± 17.5367.6 ± 52.2	−12.7 ± 0.7−11.8 ± 0.2	0.500.47
trigtacc**TCACAGC**actt**TCACAGC**tttc**TCACAGC**atctatg	wt	4.6 ± 2.7	−11.5 ± 0.2	0.30
tetragtacc**TCACAGC**actt**TCACAGC**tttc**TCACAGC**tgat**TCACAGC**atctatg	wt	2.2 ± 0.7	−14.8 ± 0.1	0.21
RD6^a^agtacc**TCACAGC**actt**TCACAGC**tttcat	PhoPC	188 ± 9	−18.9 ± 0.1	0.49
RD6-halfcagaag**TCACAGC**acttggg	PhoPC	12.2 ± 1.6μM^c^	−3.46 ± 0.42	0.62

^a^Both DR and RD6 are perfect direct-repeat sequences with a 4-bp spacer. DRs3 has the same repeated motifs but with a 3-bp spacer.

^b^N is the stoichiometry, referring to the copy number of DNA per PhoP molecule, derived from fitting of the ITC data.

^c^Binding dissociation constants of >10 μM cannot be reliably measured because of the small binding heat.
