# Supplementary material for: Structural basis of DNA sequence recognition by the response regulator PhoP in Mycobacterium tuberculosis
Source: Sci Rep. 2016 Apr 15;6:24442. doi: 10.1038/srep24442 (PMC4832192; doi:10.1038/srep24442)

Supplementary Information

Structural basis of DNA sequence recognition by the response regulator PhoP in *Mycobacterium tuberculosis*

Xiaoyuan He, Liqin Wang, and Shuishu Wang

Department of Biochemistry & Molecular Biology, Uniformed Services University of the Health Sciences, 4301 Jones Bridge Road, Bethesda, Maryland 20814, USA

Supplementary Figure S1. Superposition of the two PhoP-DNA complexes in the asymmetric unit. One of the complexes is colored green, cyan and orange, and the other is in gray. The two complexes were aligned by their effector domains. The two DNA double helices superimpose well, and the protein-DNA interfaces are identical. The receiver domains have a slight relative rotation. However, the dimer interfaces and the intrasubunit domain interfaces are identical in the two complexes.

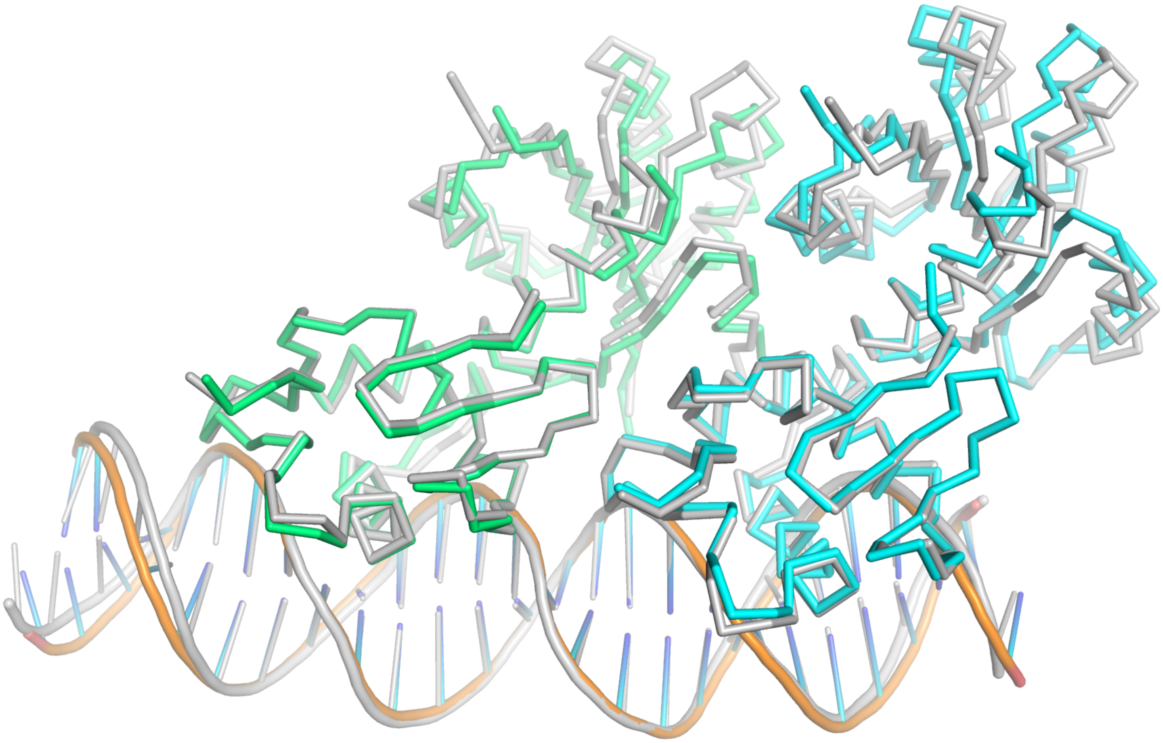

Supplementary Figure S2. Intrasubunit domain interface. The receiver domain is colored blue, and the effector domain in pale green. Side chains involved in interdomain interactions are shown as sticks. Water molecules are shown as red spheres. Hydrogen bonds are shown as yellow dashed lines. The protein main-chain atoms are not shown except the carbonyl of Phe123, which has a hydrogen bond to the amide of Tyr205. The N-terminus of α5 is next to the C-terminus of α7, contributing helix dipole interactions, a hydrogen bond between the backbone amide of Glu126 and the carbonyl of His201, and a water-mediated hydrogen bond between the backbone amide of Leu125 and the carbonyl of Asp200. The loop preceding α5 interacts with the loop following α7 through one main-chain hydrogen bond and side-chain aromatic interactions between residues Phe123 and Tyr205. The first turn of α5 interacts with α6 through charge-charge interactions between the side chain of Arg183 and those of Glu126 and Glu127, one hydrogen bond between side chains of Thr180 and Glu127, and water-mediated hydrogen bonds between Glu126 and Arg183. The C-terminal half of α5 interacts with the loop between β7 and β8 through water-mediated hydrogen bonds between Glu160 and Arg133, charge and aromatic interactions between side chains of His163 and Glu127, and hydrophobic interactions between Val134 and the hydrophobic part of Glu161.

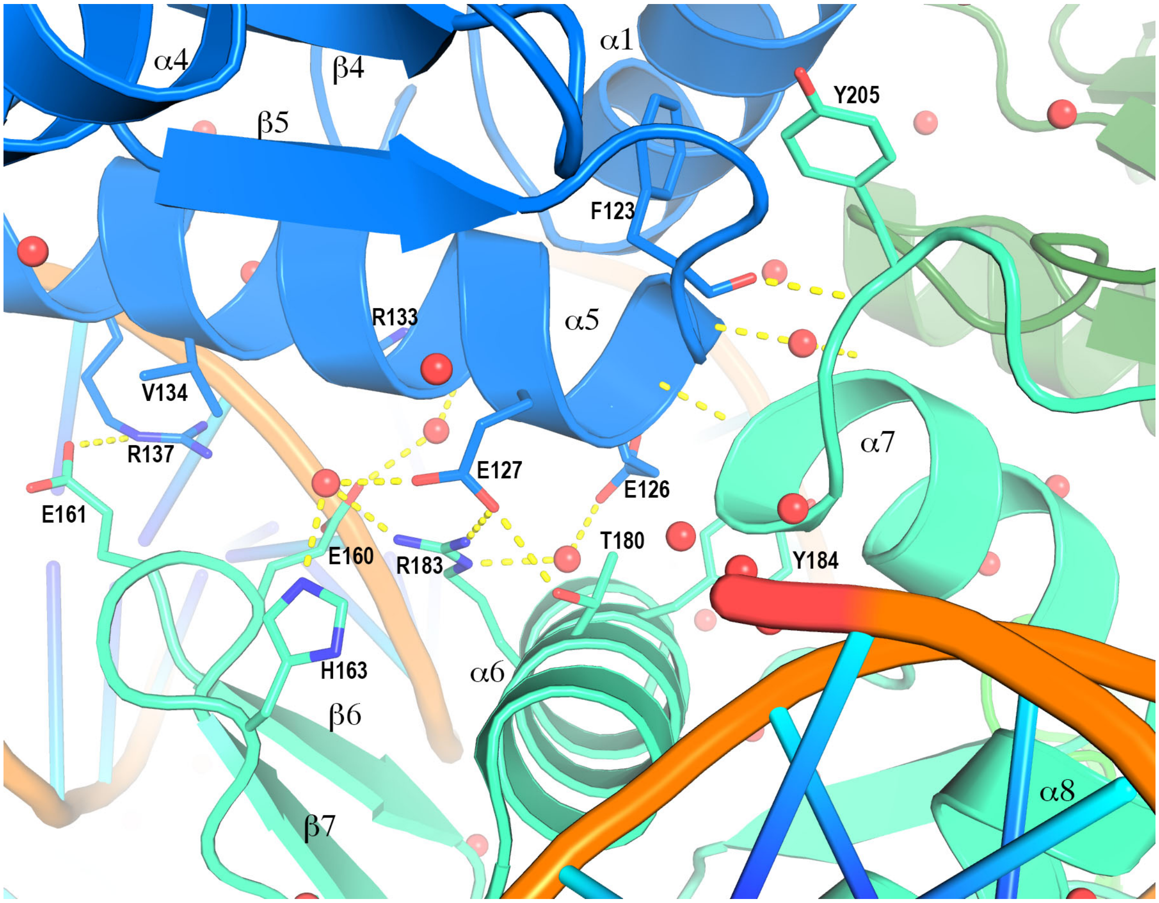

Supplementary Figure S3. Electrostatic potential surface of PhoP showing its favorable charge interactions with DNA. Molecule A of PhoP (left) is shown as electrostatic potential surface with red for the negative potential and blue for positive. The electrostatic potential was calculated by PyMOL plugin APBS and mapped onto the solvent accessible surface. Molecule B (right) is shown as ribbon diagram enclosed in a wire-mesh surface. The protein surface fits well into the adjacent major groove and minor groove surface contour of the DNA. A patch of positive electrostatic potential on the effector domain surface matches the negative charges of the DNA phosphate backbone.

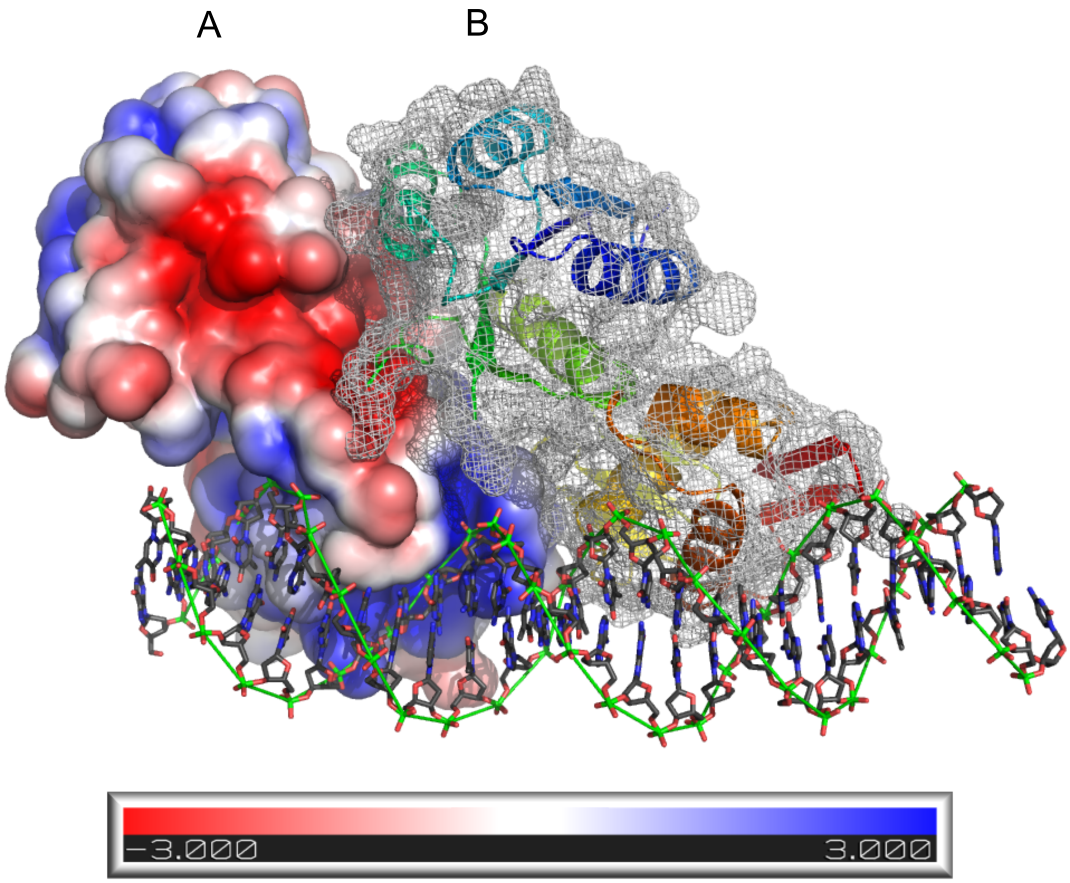

Supplementary Figure S4. Stereo diagram of initial electron density map showing continuous density for DNA. A section of the map shows the minor groove that binds the wing of the PhoP effector domain. Superimposed on the map are the refined model (colored) and the initial MR model (black). Near the center where the initial MR model is missing one base pair, the initial electron density is continuous and agrees with the refined structural model.

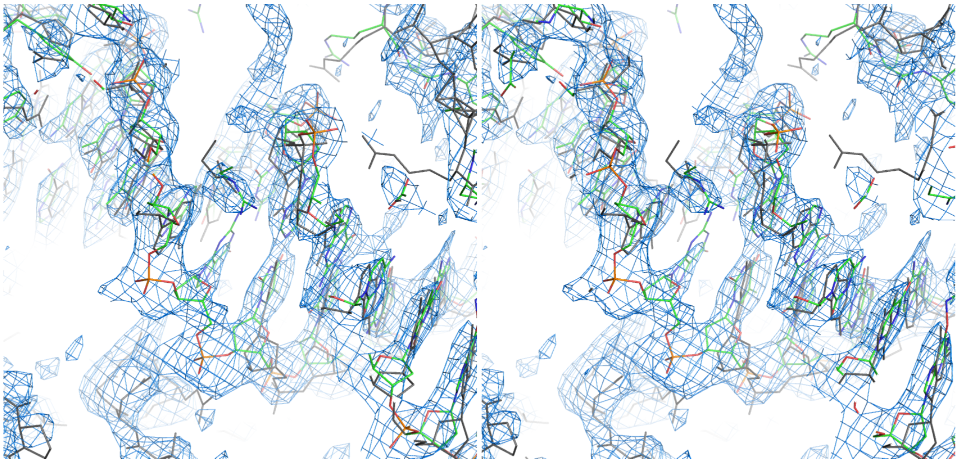

Supplementary Figure S5. Superposition of the receiver domain A of the PhoP-DNA complex with the symmetric receiver-domain dimer of the full-length PhoP structure. The PhoP-DNA complex structure is shown in green, and the symmetric receiver-domain dimer of PhoP (PDB ID 3R0J) is shown in orange. The black line represents the approximate position of the two-fold axis of the symmetric receiver-domain dimer. The receiver domains of the two structures superpose well except helices α3 and α4, which also have large shifts between the two protomers in the PhoP-DNA complex. The phosphorylation site residues are in the same positions. Slightly different side-chain conformations of these residues are due the absence of the divalent cation in the PhoP alone structure. Switch residues are in the same conformations in both structures. The side chains of L113 and L35, which are involved in the hydrophobic interaction in the tandem-dimer interface of the PhoP-DNA complex, are away from the interface of the symmetric dimer.

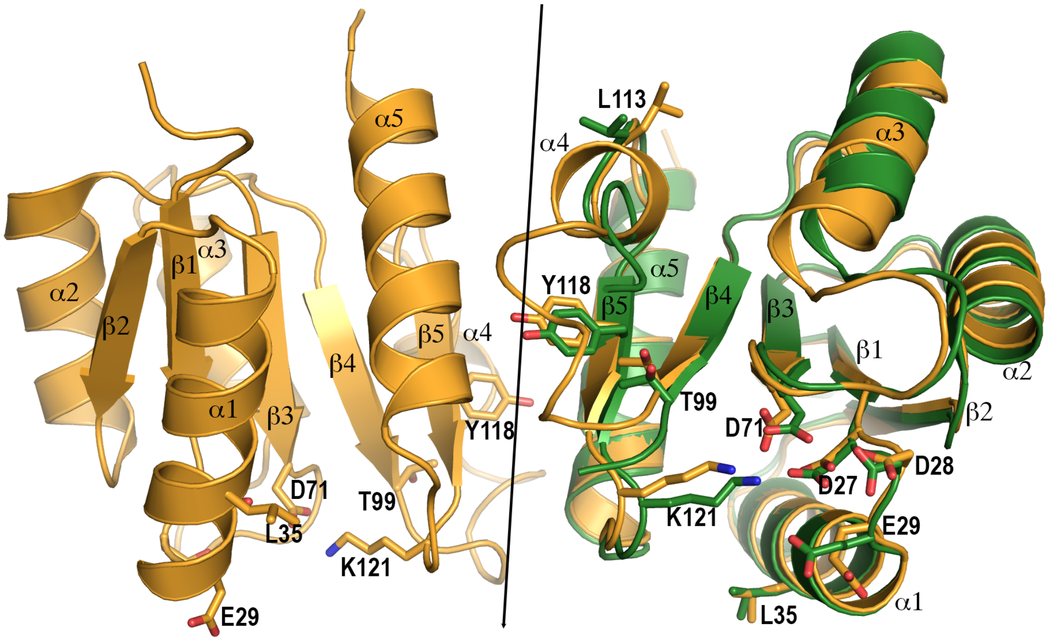

Supplement: Supplementary Information [file srep24442-s1.pdf]
